# Among the Colleges

**Published:** 1902-07

**Authors:** 


### AMONG THE COLLEGES.

Grand Rapids Veterinary College. Prospectus for this college, 1902-03, has been received, and the following changes noted in the faculty: W. W. Sammis, D.V.S., as Professor of Materia Medica and Therapeutics, in place of M. T. Banasawitz, M.D., D.V.S.; W. A. McLean, V.S., Dean, Professor of the Principles and Practice of Veterinary Medicine and Lecturer on Anatomy, in place of Coleman Nockolds, V.S., M.D.; Wm. E. Bessey, M.D., D.V.S., Professor of Comparative Physiology, in place of Charles N. Nye, D.V.S.; C. F. Haight, D.V.S., Registrar, Lecturer on Chemistry and Urinalysis, in place of A. W. Olds, Ph.C. Added: W. E. A. Wyman, M.D., V.S., as Professor of Physical Diagnosis of Lameness and Assistant to the Chair of Surgery; Allen H. Williams, M.D., as Professor of Bacteriology. Dropped: Alva Sherwood, B.S., D.V.S., Professor of Obstetrics and Assistant to the Chair of Surgery; C. E. Dornheim, Assistant Demonstrator of Anatomy; A. B. Warrener, Assistant to the Chair of Surgery.

Indiana Veterinary College. Graduating class, 1901-02: Oliver F. Campbell, Flora; William S. Eubank, Martinsville; Herbert F. Jones, Oaktown; Franklin F. Jacobs, Indianapolis, Indiana; Henry H. Lehman, Lattasburg, Ohio; Don McMahan, Noblesville; Guy C. Meskimen, Vincennes; Schuyler M. Wells, Bloomfield; Charles E. Morris, Rushville; Ondis A. Nelson, Jamestown; Herbert L. Parker, Danville; David F. Shake, Carlisle; Thomas A. Sigler, Greencastle; Mellbourne S. Tucker, Franklin; Albert H. Wacker, Indianapolis; Ira G. Wimsett, Dana; Henry O. Woodrow, Clay City, Indiana.

The eleventh annual announcement of this college for session of 1902-03 has been received, and the following changes noted: Among the officers, Alfred S. Jaeger, B.A., M.D., President, in place of Samuel E. Crose, A.M., M.D. George H. Roberts, V.S., has been added to the faculty as Professor of Cattle Pathology. There will be special Lecturers on Meat- and Milk-inspection.

---

*The Dog*, an illustrated international fortnightly, has just made its initial appearance in Philadelphia.

